# PPV May Be a Starting Point to Achieve Circulatory Protective Mechanical Ventilation

**DOI:** 10.3389/fmed.2021.745164

**Published:** 2021-12-02

**Authors:** Longxiang Su, Pan Pan, Huaiwu He, Dawei Liu, Yun Long

**Affiliations:** ^1^Department of Critical Care Medicine, State Key Laboratory of Complex Severe and Rare Diseases, Peking Union Medical College Hospital, Chinese Academy of Medical Science and Peking Union Medical College, Beijing, China; ^2^College of Pulmonary and Critical Care Medicine, Chinese PLA General Hospital, Beijing, China

**Keywords:** pulse pressure variation, mechanical ventilation, cardiopulmonary interactions, circulatory-protection, blood flow

## Abstract

Pulse pressure variation (PPV) is a mandatory index for hemodynamic monitoring during mechanical ventilation. The changes in pleural pressure (P_pl_) and transpulmonary pressure (P_L_) caused by mechanical ventilation are the basis for PPV and lead to the effect of blood flow. If the state of hypovolemia exists, the effect of the increased P_pl_ during mechanical ventilation on the right ventricular preload will mainly affect the cardiac output, resulting in a positive PPV. However, P_L_ is more influenced by the change in alveolar pressure, which produces an increase in right heart overload, resulting in high PPV. In particular, if spontaneous breathing is strong, the transvascular pressure will be extremely high, which may lead to the promotion of alveolar flooding and increased RV flow. Asynchronous breathing and mediastinal swing may damage the pulmonary circulation and right heart function. Therefore, according to the principle of PPV, a high PPV can be incorporated into the whole respiratory treatment process to monitor the mechanical ventilation cycle damage/protection regardless of the controlled ventilation or spontaneous breathing. Through the monitoring of PPV, the circulation-protective ventilation can be guided at bedside in real time by PPV.

## Highlights

- Pulse pressure variation (PPV) is a mandatory index for hemodynamic monitoring during mechanical ventilation.
- PPV originates from increased pleural pressure (P_pl_) and transpulmonary pressure (PL).
- P_pl_ mainly affects the right ventricular preload, while PL is more influenced by right heart overload.
- The role of PPV in right heart overload should be emphasized in lung and circulatory protection during mechanical ventilation, reducing the occurrence of ventilator-induced lung injury (VILI), and patient self-inflicted lung injury (P-SILI).
- PPV is a real-time monitoring index for the mechanical ventilation that can promptly alert for abnormal breathing and circulation at the bedside.

Hemodynamic monitoring is essential to improve gas exchange, optimize organ and tissue perfusion, and avoid ventilation-related lung injury (VILI) in the mechanical ventilation patients. Therefore, the low-tidal volume lung protection strategies are widely used in clinical practice. However, in the process of mechanical ventilation for the patients with acute respiratory distress syndrome (ARDS) or even coronavirus disease 2019 (COVID-19), we have increasingly realized that only low-tidal volume lung protective ventilation is far from sufficient. Many patients still die from acute circulatory failure caused by ventilation-related lung injury (VILI) or patient self-inflicted lung injury (P-SILI) (1, 2). In clinical work, we are always thinking about whether we can use a direct indicator to reflect the mechanical ventilation management to achieve circulatory protection on the basis of lung protection at the same time. The cardiopulmonary interaction produced by positive pressure ventilation leads to the changes in pleural pressure (P_pl_) and transpulmonary pressure (P_L_), and the accompanying hemodynamic effects provide clues for us, which make it possible to monitor the hemodynamic changes under mechanical ventilation (3–5). Specific indicators can prompt the hemodynamic situation of a patient to the doctor at the bedside and provide precise treatment directions. In the intensive care unit (ICU), it is necessary to establish the central venous catheters and peripheral arterial catheters for critically ill patients. The analysis of the central venous pressure, arterial blood pressure, pressure waveform, and blood gas of arteries and veins can enable doctors to perform more precise and individualized strategies in circulation and respiratory management for the patients (6). When the peripheral arterial catheter is indwelled for arterial blood pressure measurement, the arterial pressure waveform has a periodic change with the respiratory cycle that can be displayed in the monitor, which we call pulse pressure variation (PPV). The previous studies have shown that during strictly controlled mechanical ventilation, a PPV value >13% indicates that the patients have fluid responsiveness when high tidal volume or lung compliance exists (7, 8). However, sometimes we find that the PPV value is more than 13%, but the mechanical ventilation patients have no fluid responsiveness. At this time, the decline in patient cardiac output is often due to high right ventricle afterload. Therefore, expanding the usage of PPV can identify and evaluate the hemodynamic dysfunction of a patient early during mechanical ventilation and better implement circulatory and lung protection ventilation therapy.

## Mechanism of PPV

**Figure 1:**
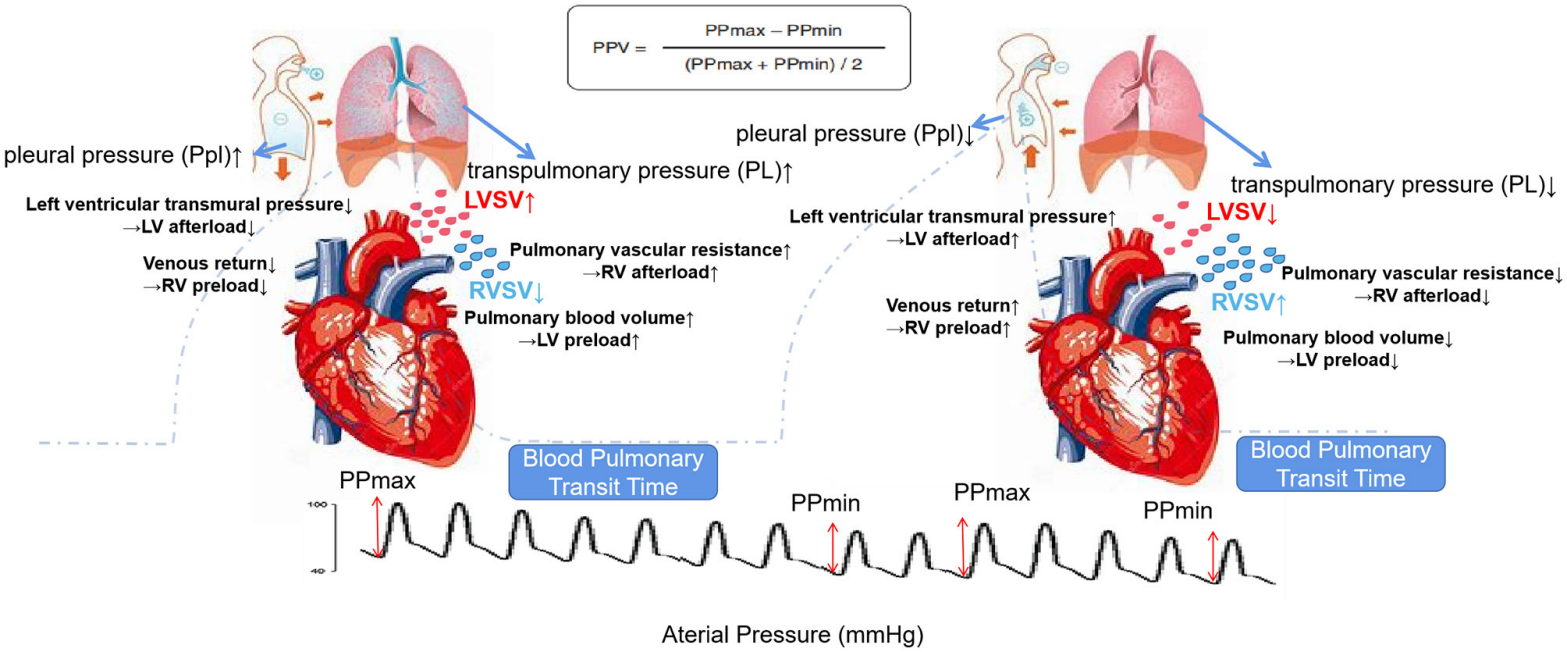
Schematic effects of high transpulmonary pressure (P_L_) and pleural pressure (P_pl_) on the right ventricle (RV) vs. left ventricle (LV) to explain the mechanisms of pulse pressure variation (PPV). Blood pulmonary transit time means 2–4 heartbeat cycles for this blood flow effect conducting from the right heart to left heart. When exhaling, the effect of the drop in the stroke volume (SV) of the right heart during inhalation will affect the left heart. Top: calculation formula of PPV; background dotted line: airway pressure tracing; bottom: arterial pressure tracing. The left side is the inspiratory phase, and the right side is the expiratory phase.

Positive-pressure ventilation changes the normal respiratory physiology of a patient, leading to the changes in P_pl_ and P_L_. The changed cardiopulmonary interaction produced by P_pl_ and P_L_ can result in the changes in preload and afterload of the two ventricles, which in turn causes the blood flow changes (3, 5). In controlled ventilation mode, inhalation can increase P_pl_ and reduce venous return, reducing the preload of the right ventricle. After the gas is inhaled, the lung tissue stress during tidal inhalation is the pressure distending the lung, called P_L_, the difference between airway pressure and P_pl_. P_L_ causes an increase in afterload to the right ventricle due to the increased pulmonary vascular resistance during inhalation (9). The result is that the output of the right ventricle is the lowest at the end of inspiration (4, 9). For the left ventricle, the increase in P_pl_ during inhalation can lead to a decrease in the afterload of the left ventricle due to the decrease in left ventricular transmural pressure. At the same time, the P_L_ causes an increase in the preload of the left ventricle. Therefore, the result for left ventricle output can be the largest at the end of inhalation (10). However, as the left and right hearts are connected, the influence of inhalation on the reduction of right ventricle output will result in the reduction of left ventricle preload. There are 2–4 heartbeat cycles for this blood flow effect conducting from the right heart to the left heart, which is called the blood pulmonary transit time. At this time, the inhalation changes to exhalation, and eventually, the left ventricle output decreases during exhalation. The different cardiac outputs of inhalation and exhalation create the basis for the existence of PPV. The maximum pulse pressure difference appears during inhalation, and the minimum pulse pressure difference appears during exhalation. The ratio of the difference between the two values to the average of the two values is defined as PPV. Figure 1 shows the schematic effects of the mechanisms of pulse pressure variation.

## PPV and Blood Flow During Mechanical Ventilation

P_pl_ and P_L_ can affect the pulmonary blood flow during mechanical ventilation. The P_pl_ causes a change in the right ventricle inflow and the left ventricle outflow. P_L_ causes the changes in right ventricle outflow and left ventricle inflow. From the above, we know that the P_pl_ during mechanical ventilation causes a decrease in the right ventricle preload, and the P_L_ causes an increase in the right ventricle afterload, both of which are responsible for the decrease in global cardiac output. However, we need to determine which pressure constitutes the bulk of decreased cardiac output so that we can better determine whether fluid resuscitation or P_L_ should be reduced. The wrong judgments will lead to the wrong treatments and ultimately cause patient injury.

In general, we focused on the hemodynamic effect of capacity status on right ventricle preload. When both the left and right ventricles have fluid responsiveness, the global cardiac output will increase as the capacity increases. At this time, the increased PPV can represent whether the patients have fluid responsiveness. If any one of the ventricles has no fluid responsiveness, the global cardiac output will ultimately not increase due to fluid resuscitation (10). When pulmonary compliance is normal or slightly decreased, a high tidal volume can cause P_pl_ to affect the change in alveolar pressure by 50% or more. At this time, if combined with a hypovolemic state, the influence of increased P_pl_ on right ventricle preload will constitute the bulk of cardiac output, resulting in a positive PPV (11). Therefore, when using PPV to predict the fluid responsiveness during mechanical ventilation, the doctors need to consider pulmonary compliance and tidal volume (7, 12).

In addition, PPV can be used to predict the hemodynamic effect of positive end-expiratory pressure (PEEP) on the right ventricle (13). High PEEP can cause decreased cardiac output, resulting in increased PPV, but not all the patients have fluid responsiveness. Research has suggested that the higher the PPV before using mechanical ventilation, the more obvious the decrease in cardiac output caused by PEEP (13). However, for the patients without fluid responsiveness, if the high PPV does not represent reduced right ventricle preload, we need to consider whether it is due to the increased right ventricle afterload that causes the decrease in cardiac output. This suggests that the high PPV in the patients with ARDS may not mean hypovolemia but a significant increase in P_L_, which causes an increase in right ventricle afterload and a decreased left ventricle inflow. At this time, we should not only take note of cardiac output decreases but also be alerted of the incidence of acute cor pulmonale (ACP).

## PPV and Right Ventricle Afterload

Positive pressure ventilation affects the right ventricle afterload, which in turn affects pulmonary blood flow, resulting in a decrease in cardiac output and an increase in PPV. At this time, the patients usually have no fluid responsiveness. P_L_ rises during mechanical ventilation, which can compress the alveolar capillaries. Since right heart failure is more sensitive to afterload than preload, the decrease in right ventricular outflow during inhalation has a greater relationship with right ventricle afterload. Two clinical studies have reported that in the case of right heart failure, the patients with high PPV have no fluid responsiveness. Moritz et al. found that in the early stage after cardiac surgery or during septic shock, the patients with increased pulmonary artery pressure had a poor response to fluid resuscitation, and PPV could not be used to predict the fluid responsiveness (14). Yazine Mahjoub et al. (15) found that right heart failure can lead to false-positive PPV. Such patients can be combined with the tricuspid annular systolic velocity (Sta) to identify whether the patients have increased right ventricular afterload. Therefore, we emphasize that when the capacity of a patient is sufficient but lung compliance is significantly reduced or when the ventilator support conditions are high, P_L_ can significantly increase the right ventricular afterload or cause ACP, resulting in a decrease in cardiac output (3). As mentioned before, the effects of mechanical ventilation on preload and afterload of the right ventricle can cause cardiac output and blood pressure to decrease, leading to an increase in PPV, but different effects will have completely different treatments. When decreased right ventricular preload contributes to the bulk of decreased cardiac output, it indicates the need for fluid resuscitation; when the right ventricular afterload effect is dominant, fluid resuscitation is not recommended or may even be harmful. In this case, limiting P_L_ is the first choice, that is, the essence and connotation of circulation-protective ventilation.

## PPV and Spontaneous Breathing

Many studies have recommended reducing the dose of sedatives during mechanical ventilation and allowing the patients to maintain spontaneous breathing. However, the abnormal spontaneous breathing of the mechanically ventilated patients, defined here as excessive spontaneous breathing, can cause hemodynamic disturbances. Excessive spontaneous breathing causes negative changes in the Ppl and increases the P_L_, resulting in excessive alveolar expansion, increased functional residual capacity (FRC), increased pulmonary vascular resistance (PVR), decreased pulmonary blood flow, increased V/Q ratio, and increased dead space ventilation (16). The injury caused by spontaneous breathing not only affects the alveoli but also affects pulmonary blood flow. Transvascular pressure is the difference between intravascular pressure and pressure outside the vessels. The more negative P_pl_ generated during spontaneous breathing will increase transvascular pressure and aggravate pulmonary edema due to promoting alveolar flooding and increased RV flow (17). In the healthy lung, the changes in local P_pl_ are evenly transmitted across the lung surface; this phenomenon is called “fluid-like” behavior. However, the injured lungs exhibit “solid-like” behavior, where a non-aerated lung region impedes the rapid generalization of a local change in P_L_. In such cases, the lung expansion is heterogeneous. Therefore, there will be different regional P_Ls_ and different degrees of lung inflation. At this time, if spontaneous breathing is superimposed, it will undoubtedly cause more changes in P_pl_ and uneven conduction, resulting in uneven lung expansion or even excessive expansion (18, 19). Eventually, lack of synchronization between the reserved spontaneous breathing and mechanical ventilation of a patient as well as mediastinal swing may impair pulmonary circulation and right heart function (20, 21). The hemodynamic effects caused by the above conditions can also be predicted by PPV.

## PPV And COVID-19 AND ARDS

In COVID-19 pneumonia, a large proportion of the patients have L-type ARDS. Their lung gas volume is usually high, lung recruitability is minimal, and hypoxemia likely results from the loss of hypoxic pulmonary vasoconstriction and impaired regulation of pulmonary blood flow (22). During hypoxia, the patients are usually forced to increase their tidal volume and thus increase minute ventilation volume (23). At this time, if the patients maintain spontaneous breathing, there will be an increase in the work of spontaneous breathing, which will cause the P_pl_ negative changes and increase the transvascular pressure. In the case of increased pulmonary vessel permeability caused by inflammation, increased transvascular pressure will extremely increase the risk of pulmonary edema resulting from vascular leakage (24), which will progress L-type ARDS toward H-type ARDS. Manifestation of H-type is similar to typical ARDS. The lung lesions are distributed in a dependent area. The movement of the diaphragm during spontaneous breathing is mainly in the dependent area, which is conducive to the recruitment maneuvers and increased pulmonary blood flow in the dependent area (25–27). When mild and moderate ARDS occurs, the patients have a relatively low P_L_, and spontaneous breathing can increase transpulmonary vascular pressure, reduce pulmonary vascular resistance, increase pulmonary blood flow, and improve ventilation while improving oxygenation. However, in severe ARDS, the patients have higher P_L_, spontaneous breathing can cause uneven blood flow and airflow distribution, alveoli easily expand excessively, pulmonary vascular resistance increases and decreases pulmonary blood flow in normal ventilation areas, and transpulmonary vascular pressure increases and exacerbates pulmonary edema in dependent areas. Unfortunately, we have not yet seen the publication of studies on the treatment or prognosis of PPV in the patients with COVID-19 until now. However, according to the principle of PPV, a high PPV plays an important role in the monitoring of circulation damage/protection during mechanical ventilation. We hypothesize that the PPV abnormalities in the patients with COVID-19 may be a common phenomenon. Through PPV monitoring, the circulation management of mechanical ventilation can be incorporated into the entire mechanical ventilation and weaning process.

## Limitations of PPV

When PPV is used as an index to predict fluid responsiveness, we need to clarify whether PPV is positive. We must also be alert to the possibility of false negatives and positives and the corresponding reasons. Multiple studies have shown that the threshold index for PPV to assess fluid responsiveness is 13% (8, 28). In the clinical applications, it should be noted that when the tidal volume is >8 ml/kg tidal volume (VT) mechanical ventilation, the PPV fluctuates at “9% < PPV ≤ 13%” and may also prompt the possibility of fluid responsiveness (29). Myatra et al. proposed that PPV is poor in predicting fluid responsiveness at 6 ml/kg VT (AUROC curve, 0.69). When VT increased to 8 ml/kg, PPV more reliably predicted fluid responsiveness (AUROC curve, 0.91). The study also found that during the increase in VT, if the absolute value of PPV increases by ≥3.5%, the fluid responsiveness can be predicted quite accurately (AUROC curve, 0.99) (30). Messina et al. (31) conducted a tidal volume challenge (TVC) in the prone position patients, that is, increasing the tidal volume from 6 to 8 ml/kg in a short period of time, and attempting to use pulse pressure variability and stroke volume variability to assess the fluid responsiveness of a patient. In addition, the studies have pointed out that when the PPV of a patient is >13%, the passive leg raising tests are performed. If the PPV decreases, it indicates that the patient has fluid responsiveness. If the PPV is unchanged, it indicates that the right ventricle afterload of a patient increases (32). Therefore, we must pay attention to the premise of ensuring tidal volume (8 ml/kg PBW) when we use PPV to judge potential fluid responsiveness. Additionally, we can combine or use more indicators for judging fluid responsiveness, for example, transmural central vascular pressure, to further improve the accuracy of PPV (33, 34). However, we may not need to emphasize whether the patient is in a fully controlled ventilation state or whether the tidal volume needs to be increased to 8 ml/kg when we use PPV as an indicator of right ventricle afterload. What we need to pay attention to is whether P_L_ affects the pulmonary circulation and right heart function. However, the only concern is to identify whether the patient has arrhythmia. Arrhythmia has become the most important obstacle that affects the accurate determination of PPV. In addition, we should also notice the effect of increased intra-abdominal pressure on P_pl_ and P_L_. In general, the clinical significance of positive PPV has a new extension and significance than before.

## Circulation-Protective Ventilation Strategy

**Figure 2:**
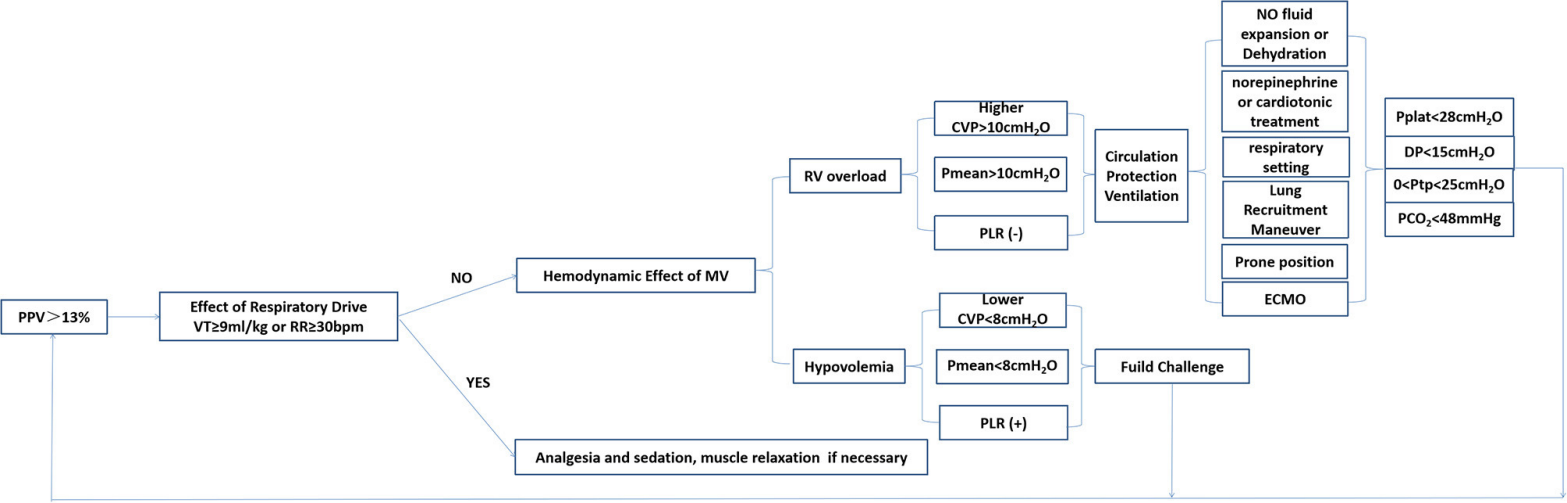
Management and intervention process based on PPV monitoring. PPV, pulse pressure variation; MV, mechanical ventilation; RV, right ventricle; CVP, central venous pressure; P_mean_, mean airway pressure; PLR, passive leg raising test; ECMO, extracorporeal membrane oxygenation; DP, driving pressure; Ptp, transpulmonary pressure.

At present, the lung-protection ventilation strategies have been affirmed and widely used, such as low tidal volume, limiting plateau pressure (<27 cmH_2_O), and driving pressure (<15 cmH_2_O), to reduce lung stress injury caused by mechanical ventilation. Recruitment maneuver and perform reasonable PEEP titration can also be considered. Applying P_L_ monitoring achieves the end-expiratory P_L_ > 0 cmH_2_O and end-inspiratory P_L_ <25 cmH_2_O. As high PaCO_2_ can aggravate pulmonary vasoconstriction, treatment should be used to limit hypercapnia (PaCO_2_ <48 mmHg). Prone position ventilation is used to reduce pulmonary circulation resistance, improve ventilation and blood flow matching, and improve right heart function. Therefore, based on the above treatment to prevent secondary injury (35), what else can we do? A new understanding of PPV will help us to protect the circulation during mechanical ventilation. After the relevant circulation-protective ventilation treatment, the criterion for whether we successfully protect circulation is whether the PPV returns to normal. Therefore, in the process of related lung protection ventilation, the following circulation-protective procedures can be considered to guide our mechanical ventilation to achieve lower PPV, such as evaluating and treating the spontaneous breathing effort and respiratory support conditions and monitoring and optimizing the flow status and volume status of a patient. Combined with our previous studies (36, 37), we propose a possible processing procedure for the PPV abnormalities, as shown in Figure 2.

In summary, we should pay attention to the role of P_pl_ and P_L_ in the cardiopulmonary interaction and their effect on blood flow. We should be sufficiently aware of the universality of increased right ventricle afterload caused by mechanical ventilation. PPV is an indicator that can help us distinguish the causes of decreased cardiac output and has become a necessary indicator for monitoring circulatory damage in ARDS during mechanical ventilation. At the bedside, we can adjust our mechanical ventilation parameter by monitoring the PPV to achieve circulation-protective mechanical ventilation. Therefore, we believe that PPV is the starting point to achieve circulation-protective mechanical ventilation. During mechanical ventilation, PPV should be continuously monitored, and its abnormalities should be treated clinically and dealt with immediately instead of shelving.

## Author Contributions

PP, LS, and HH wrote the manuscript. DL and YL responsible to this manuscript. All authors contributed to the article and approved the submitted version.

## Funding

This study was supported by the Beijing Nova Program (Z201100006820126) from the Beijing Municipal Science and Technology Commission and Capital Characteristic Clinic Project of Beijing (No. Z181100001718209), Excellence Program of Key Clinical Specialty of Beijing for critical care medicine in 2020 (ZK128001), Beijing Municipal Science and Technology Commission (No. Z201100005520051), and Capital's Funds for Health Improvement and Research (No. 2020-2-40111).

## Conflict of Interest

The authors declare that the research was conducted in the absence of any commercial or financial relationships that could be construed as a potential conflict of interest.

## Publisher's Note

All claims expressed in this article are solely those of the authors and do not necessarily represent those of their affiliated organizations, or those of the publisher, the editors and the reviewers. Any product that may be evaluated in this article, or claim that may be made by its manufacturer, is not guaranteed or endorsed by the publisher.
